# The Running Wheel Enhances Food Anticipatory Activity: An Exploratory Study

**DOI:** 10.3389/fnbeh.2016.00143

**Published:** 2016-07-05

**Authors:** Danilo E. F. L. Flôres, Crystal N. Bettilyon, Lori Jia, Shin Yamazaki

**Affiliations:** ^1^Department of Neuroscience, University of Texas Southwestern Medical CenterDallas, TX, USA; ^2^Institute of Biosciences, University of São PauloSão Paulo, Brazil; ^3^Hockaday SchoolDallas, TX, USA

**Keywords:** circadian, mouse, reward, restricted feeding, food-entrainable oscillator

## Abstract

Rodents anticipate rewarding stimuli such as daily meals, mates, and stimulant drugs. When a single meal is provided daily at a fixed time of day, an increase in activity, known as food anticipatory activity (FAA), occurs several hours before feeding time. The factors affecting the expression of FAA have not been well-studied. Understanding these factors may provide clues to the undiscovered anatomical substrates of food entrainment. In this study we determined whether wheel-running activity, which is also rewarding to rodents, modulated the robustness of FAA. We found that access to a freely rotating wheel enhanced the robustness of FAA. This enhancement was lost when the wheel was removed. In addition, while prior exposure to a running wheel alone did not enhance FAA, the presence of a locked wheel did enhance FAA as long as mice had previously run in the wheel. Together, these data suggest that FAA, like wheel-running activity, is influenced by reward signaling.

## Introduction

Organisms use circadian clocks to anticipate daily changes in the environment (temperature, food availability, and predation). It has been suggested that this anticipation is critical for survival (Antle and Silver, 2009; DeCoursey, 2014; Spoelstra et al., 2016). For proper anticipation clocks must retain a stable phase-relationship with, or entrain to, daily environmental cycles (Winfree, 1980; Johnson et al., 2003). Light is the primary environmental signal that entrains a master circadian pacemaker located in the suprachiasmatic nucleus (SCN; Moore and Eichler, 1972; Stephan and Zucker, 1972; Rusak, 1977). The SCN orchestrates an ensemble of rhythms among the peripheral circadian clocks located in most peripheral organs (Yamazaki et al., 2000; Yoo et al., 2004; Izumo et al., 2014) and controls circadian rhythms in physiology and behavior (Moore and Eichler, 1972; Sawaki et al., 1984; Lehman et al., 1987; Ralph et al., 1990). The molecular machinery that generates these circadian rhythms in the SCN and peripheral clocks has been extensively characterized (Ko and Takahashi, 2006). Daily food availability is another signal that can entrain circadian clocks. In laboratory studies, locomotor activity of rodents entrains to restricted food availability, and the rodents become active several hours before food is presented (Richter, 1922). This so-called food anticipatory activity (FAA) is controlled by a circadian pacemaker, the food-entrainable oscillator (Mistlberger, 1994; Stephan, 2002). SCN-ablated animals still exhibit normal FAA, indicating that the food-entrainable oscillator is located outside of the SCN (Mistlberger, 1994; Stephan, 2002). However, despite exhaustive attempts to identify the food-entrainable oscillator, its anatomical locus has yet to be discovered (Davidson, 2009). It has also been shown that the food-entrainable oscillator does not rely on the canonical molecular circadian timekeeping mechanism (Pitts et al., 2003; Iijima et al., 2005; Pendergast et al., 2009, 2012; Storch and Weitz, 2009; Flores et al., 2016). Although the circadian properties of food entrainment have been elucidated, the factors affecting the expression of FAA have not been well-studied. Understanding factors that regulate the robustness of FAA may provide clues toward identifying the anatomical locus of the food-entrainable circadian oscillator.

Several lines of evidence suggest the reward system is involved in FAA expression (Webb et al., 2009). In addition to anticipating restricted food availability, rodents also anticipate other daily rewarding stimuli, such as palatable meals, females in estrous, and stimulant drugs (Mistlberger and Rusak, 1987; Mendoza et al., 2005a,c; Verwey et al., 2007; Angeles-Castellanos et al., 2008; Hsu et al., 2010a,b; Jansen et al., 2012; Landry et al., 2012; Keith et al., 2013; Mohawk et al., 2013; Flores et al., 2016). Recent studies show that dopamine and opioids, which are important components of the reward pathway, regulate the robustness of FAA (Kas et al., 2004; Mendoza and Challet, 2014). FAA is attenuated in D1 receptor, but not D2 receptor, knockout mice (Gallardo et al., 2014; Michalik et al., 2015). Systemic pre-treatment (before the onset of food) with D1 and D2 receptor antagonists attenuated FAA in ICR mice (Liu et al., 2012). It has also been shown that mu-opioid receptor knockout mice show diminished FAA (Kas et al., 2004). Together these data suggest that dopamine and endogenous opioids can alter the robustness of FAA. Wheel-running is rewarding to rodents and is known to activate the dopaminergic system and influence endogenous opioids in their brains (Sherwin, 1998; Novak et al., 2012; Morgan et al., 2015). In this study, we investigate the effect of the running wheel on the robustness of FAA in C57BL/6J mice.

## Materials and Methods

### Animals

Wild-type C57BL/6J male mice (*n* = 27, 4–10 weeks of age) were obtained from either the E. K. Wakeland Mouse Breeding Core (UT Southwestern, Dallas, TX, USA) or our breeding colony at UT Southwestern. After weaning, mice were group-housed with *ad libitum* access to chow (Teklad Global 18% Protein Rodent Diet 2918; Harlan, Madison, WI, USA) and water in cages without running wheels. The 12 h light:12 h dark (12L:12D) cycle in the holding room was generated by fluorescent bulbs. All experiments were carried out in accordance with the National Institutes of Health Guidelines regarding the care and use of animals for experimental procedures and were approved by the Institutional Animal Care and Use Committee at UT Southwestern Medical Center (Protocol #: 2013-0035).

### Activity Recording

Animals were singly housed in cages with *ad libitum* access to water in light-tight ventilated boxes (22–23°C, 19–54% relative humidity) in 18L:6D or 12L:12D. Light was generated by white LEDs (40 μW/cm^2^/s, 220 lux inside the cage) or green LEDs (7 μW/cm^2^/s, 55 lux inside the cage); specified for each experiment (see figure legends). Mice were housed either in cages with running wheels (length × width × height: 29.5 × 11.5 × 12.0 cm; wheel diameter 11.0 cm) or without running wheels (29.5 × 11.5 × 12.0 cm or 28.5 × 16.5 × 13.0 cm). In experiments with locked wheels, a clip was used to prevent the wheel from rotating. The number of wheel revolutions was monitored by a micro-switch and general activity was monitored with a passive infrared sensor (product ID 189, Adafruit, New York City, NY, USA) placed above the cage. Activity was continuously recorded every minute using the ClockLab system (Actimetrics, Wilmette, IL, USA). Cages and water bottles were changed at least once every 3 weeks.

### Restricted Feeding

In both 18L:6D and 12L:12D, food was initially removed 2 h before lights-off and left out for 16 h. For 2 days, chow was provided for 8 h (from 10 h before lights-off to 2 h before lights-off), and then for 6 h (from 10 h before light-off to 4 h before lights-off) for the subsequent 2 days. Thereafter, chow was provided for 4 h (from 8 h before lights-off to 4 h before lights-off). Food was manually placed on the bottom of the cage and/or in the food hopper. When food was removed, the bottom of the cage was carefully inspected to remove any remaining small pieces of chow.

### Data Analysis

Activity was double-plotted in actograms (6-min bins; normalized format, ClockLab). Actograms (wheel-running and general activity) for all individual mice are provided in the Supplementary Material. Twenty-four hour individual average activity profiles were used to quantify the robustness of FAA. Using ClockLab, individual average activity profiles (6-min bins) were generated from activity data for the last 5 or 7 days (specified in the figure legends) in each wheel condition. The robustness of FAA was quantified by measuring the area under the curve of the bout of activity that occurred prior to the onset of food availability (Supplementary Figure 1). The area under the curve was measured by free-hand tracing using the ImageJ software (National Institutes of Health). The twenty-four hour group average profiles are the average of the individual activity profiles.

### Statistics

The summary of the statistics used is presented in Supplementary Table 1. The robustness of FAA (FAA AUC) was compared among the different cage types in each experiment. Because the experimental conditions in each experiment were slightly different, we did not compare FAA between experiments. The data for each cage type was tested for normality using the Shapiro-Wilk test. When data had no normal distribution, a non-parametric test was used. Either the paired two-tailed *t*-test or Wilcoxon signed-rank test was used for experiments with two conditions. ANOVA with repeated measures was used for experiments with three conditions, and if significance was detected a *post hoc* multiple comparison test (Tukey’s) was used. The FAA AUC measure for animal #206 in the Locked Wheel cage (2757) was identified beyond the Quartile 3 (Q_3_) + 1.5 × Interquartile Range (IQR). Therefore, #206 was excluded from the statistical analysis. The software PAST version 3.08 (Hammer et al., 2001) was used for all statistical analyses. Criteria (*p* < 0.05) was used for statistical significance.

## Results

### Wheel-Running Enhances Food Anticipatory Activity

**Figure 1:**
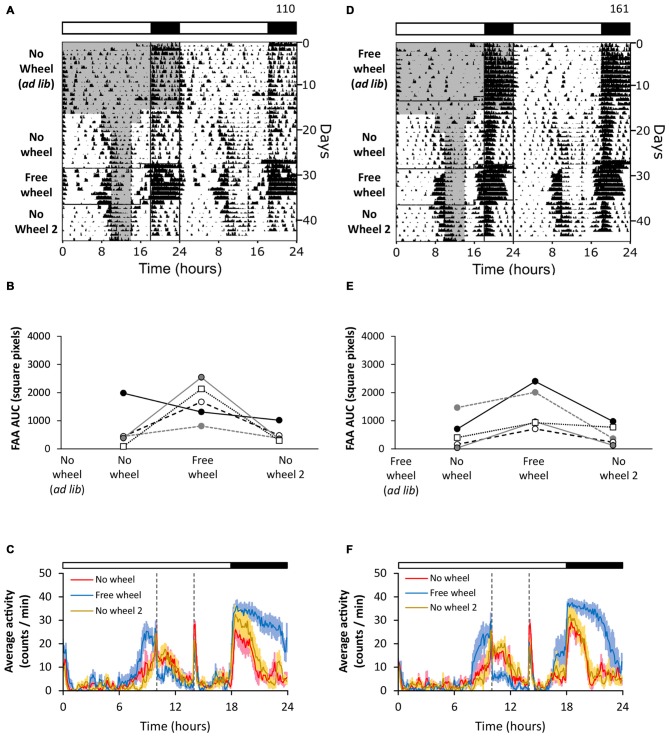
**Wheel-running enhances food anticipatory activity (FAA) irrespective of prior wheel running experience.** FAA from wheel-naïve (**A–C**; *n* = 5) and wheel-exposed (**D–F**; *n* = 5) mice was measured in 18L:6D (white LEDs). During restricted feeding, chow was manually placed and removed from the food hopper. The times when food was available are shown as gray shading on the left half of each representative double-plotted actogram (**A,D**; white and black bars show light and dark, respectively). FAA was quantified (AUC of the last 5 days of data) for each mouse (**B,E**; unique symbols connected by lines) in successive no-wheel, free-wheel, and then no-wheel conditions. Twenty-four hour group average activity profiles (**C,F**; dotted vertical lines indicate the time of food availability) show the mean (solid line) and standard deviation (light colored area). Time 0 is lights on. Cage dimensions: no-wheel: 29.5 × 11.5 × 12.0 cm; free-wheel: 29.5 × 11.5 × 12.0 cm with an 11.0 cm diameter running wheel. All individual actograms and activity profiles are shown in the Supplementary Figures 2–4.

We first determined whether wheel running activity affects FAA (Figures 1A–C). We housed mice in cages without running wheels (Figure 1A, no wheel *ad libitum*) and then performed daytime restricted feeding. Weak FAA was present during restricted feeding without a running wheel (Figures 1A–C, no wheel). After 8 days of stable 4 h restricted feeding, we moved mice to cages with running wheels and continued restricted feeding. The robustness of FAA was increased by the introduction of the running wheel (Figures 1A–C, free wheel; except one mouse shown in Figure 1B). The effect of the running wheel extinguished upon moving the mice to cages without running wheels, evidenced by the decrease in FAA robustness back to pre-wheel levels (Figures 1A–C, no wheel 2). These data show that the robustness of FAA was enhanced by running wheel activity, but this enhancement was lost when the wheel was removed.

### Prior Wheel-Running Experience Alone does not Enhance FAA

We next determined whether prior wheel-running experience affected the robustness of FAA (Figures 1D–F). We first housed mice in cages with running wheels and provided chow *ad libitum* (Figure 1D, free wheel *ad libitum*). On day 13, we moved the mice to cages without wheels and performed restricted feeding (Figures 1D–F, no wheel). The mice expressed weak FAA that was enhanced when the mice were subsequently moved to cages with running wheels (Figures 1D–F, free wheel). Then, the robustness of FAA decreased again when the mice were moved back to cages without wheels (Figures 1D–F, no wheel 2). This study shows that prior experience of wheel-running alone did not enhance FAA.

### Naïve Exposure to a Locked Running Wheel Alone does not Enhance FAA

**Figure 2:**
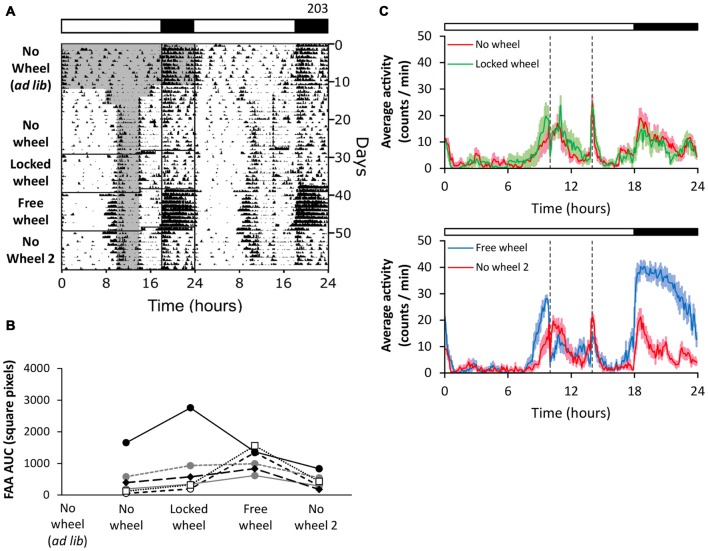
**Naïve exposure to a locked running wheel alone does not enhance FAA.** FAA from wheel-naïve mice (*n* = 6) was measured in 18L:6D (green LEDs). During restricted feeding, chow was manually placed and removed from the food hopper and cage bottom. The times when food was available are shown as gray shading on the left half of the representative double-plotted actogram (**A**; white and black bars show light and dark, respectively). FAA was quantified (AUC of the last 7 days of data) for each mouse (**B**; unique symbols connected by lines) in successive no-wheel, locked-wheel, free-wheel, and no-wheel conditions. Twenty-four hour group average activity profiles (**C**; dotted vertical lines indicate the time of food availability) show the mean (solid line) and standard deviation (light colored area). Time 0 is lights on. Cage dimensions: no-wheel: 28.5 × 16.5 × 13.0 cm; with-wheel (locked and free): 29.5 × 11.5 × 12.0 cm with an 11.0 cm diameter running wheel. All individual actograms and activity profiles are shown in the Supplementary Figures 5–7.

We next tested if the presence of a locked wheel in the cage enhances FAA (Figure 2). Consistent with our previous results, when we performed restricted feeding in mice housed in cages without wheels, the mice had weak FAA (Figure 2, no wheel). On day 29, we moved the mice to cages with locked wheels and continued restricted feeding. In the presence of the locked wheel, FAA remained weak (Figure 2, locked wheel). To determine if the free wheel had an enhancement effect on this cohort, we next unlocked the wheels (Figure 2, free wheel). As before, the robustness of FAA was enhanced with rotating running wheels, and FAA returned to low levels (similar to the locked wheel condition) when the wheels were removed (Figure 2, no wheel 2). These data demonstrate that the presence of a locked running wheel alone did not enhance FAA.

### Prior Wheel-Running Experience Combined with a Locked Wheel Enhances FAA

**Figure 3:**
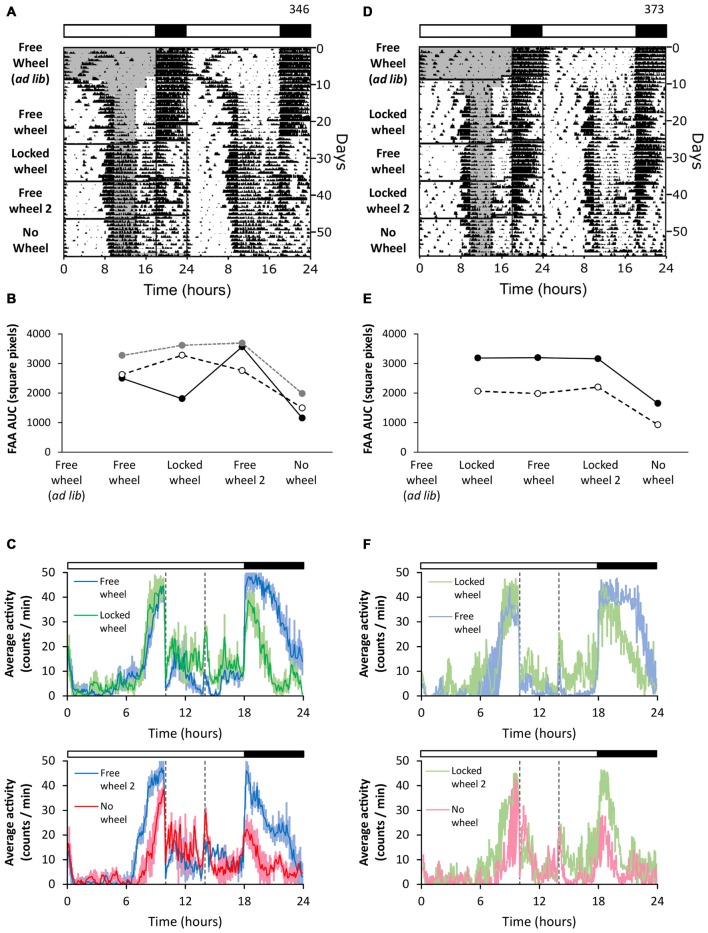
**Prior wheel-running experience combined with a locked wheel enhances FAA.** Mice were first exposed to running wheels in 18L:6D (green LEDs). Restricted feeding was initiated in either free-wheel (**A–C**; *n* = 3) or locked-wheel (**D–F**; *n* = 2) conditions. During restricted feeding, chow was manually placed and removed from the food hopper and cage bottom. In **(B)**, FAA was quantified (AUC of the last 7 days of data) for each mouse (unique symbols connected by lines) in successive free-wheel, locked-wheel, free-wheel, and no-wheel conditions. In **(E)**, FAA was quantified (AUC of the last 7 days of data) for each mouse (unique symbols connected by lines) in successive locked-wheel, free-wheel, locked-wheel, no-wheel conditions. Twenty-four hour group average activity profiles (**C,F**; dotted vertical lines indicate the time of food availability) show the mean (solid line) and standard deviation **(C)** or range **(F)** with light colored areas. Time 0 is lights on. Cage dimensions: no-wheel: 28.5 × 16.5 × 13.0 cm; with-wheel (locked and free): 29.5 × 11.5 × 12.0 cm with an 11.0 cm diameter running wheel. All individual actograms and activity profiles are shown in the Supplementary Figures 8–10.

Next we tested if previous wheel-running experience affected FAA in the presence of a locked wheel (Figure 3). We housed mice with freely rotating wheels (Figures 3A–C, free wheel *ad libitum*) and performed restricted feeding (Figures 3A–C, free wheel). The mice developed robust FAA. When the wheels were locked, the robustness of FAA was unchanged (Figures 3A–C, locked wheel). FAA stayed robust when the wheels were unlocked again (Figures 3A–C, free wheel 2). Interestingly, though, after an intervening exposure to free wheel (and enhancement of FAA), mice were placed in cages with no wheels and the robustness of FAA decreased to levels lower than the locked wheel condition (Figures 3A–C, no wheel). These data demonstrate that after prior wheel-running, locked wheels were able to enhance the robustness of FAA.

In the previous experiment, FAA was established in the presence of freely rotating wheels. Next we exposed mice to free wheels during *ad libitum* feeding (Figure 3D, free-wheel *ad libitum*), but then started restricted feeding after the running wheels were locked (Figures 3D–F, locked wheel). The mice developed robust FAA in this paradigm even though restricted feeding was performed only in the presence of locked wheels. When wheels were then unlocked, FAA remained robust but did not increase further (Figures 3D–F, free wheel). FAA remained robust upon locking the wheels again (Figures 3D–F, locked wheel 2). In contrast, when the mice were subsequently moved to cages without wheels, the robustness of FAA decreased (Figures 3D–F, no wheel). Together these data show that locked wheels enhanced FAA, as long as the mice previously participated in wheel-running activity. These findings should be verified in future studies with additional mice.

### Photoperiod Alters the Robustness of FAA

**Figure 4:**
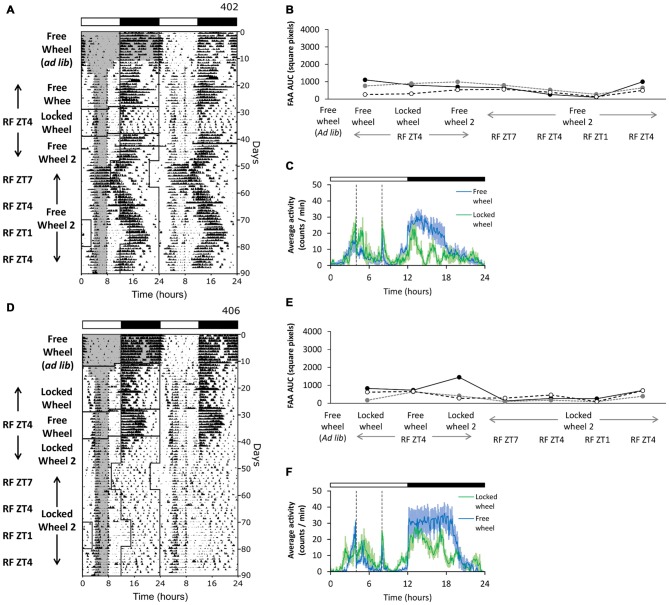
**Photoperiod alters the robustness of FAA.** Mice were first housed with freely rotating wheels in 12L:12D (green LEDs). Restricted feeding was initiated in either free-wheel (**A–C**; *n* = 3) or locked-wheel (**D–F**; *n* = 3) conditions. During restricted feeding, chow was manually placed and removed from the food hopper and cage bottom. In **(B)**, FAA (food available ZT4–8) was quantified (AUC of the last 7 days of data) for each mouse (unique symbols connected by lines) in successive free-wheel, locked-wheel, and free-wheel conditions. In **(E)**, FAA was quantified (AUC of the last 7 days of data) for each mouse (unique symbols connected by lines) in successive locked-wheel, free-wheel, and locked-wheel conditions. Then, the mice remained in their respective free-wheel **(A,B)** or locked-wheel **(D,E)** conditions and the LD cycle was first advanced 3 h (feeding ZT7–11), then delayed 3 h (ZT4–8), then delayed another 3 h (ZT1–5), and finally advanced 3 h (ZT4–8). Cage dimensions: with-wheel (locked and free): 29.5 × 11.5 × 12.0 cm with an 11.0 cm diameter running wheel. All individual actograms and activity profiles are shown in the Supplementary Figures 11–13.

Previous studies have shown that dopamine signaling is influenced by photoperiod (Sorg et al., 2011; Deats et al., 2015; Goda et al., 2015). We previously found that FAA is more robust in long photoperiods (18L:6D) compared to short photoperiods (12L:12D) (Pendergast et al., 2009). To test whether there was a synergistic effect of long photoperiod and running wheel activity on the robustness of FAA, we performed restricted feeding in 12L:12D to contrast our results in 18L:6D. All mice were initially housed with free wheels and fed *ad libitum* in 12L:12D (Figures 4A,D, free wheel *ad libitum*). We then gave the mice either free wheels or locked wheels and performed restricted feeding in 12L:12D (Figure 4, free wheel, locked wheel). Mice in both conditions developed FAA. Because all mice initially participated in wheel-running activity, the robustness of FAA was similar in mice with free wheels and locked wheels (Figures 4A–F). However, FAA was less robust in 12L:12D compared to 18L:6D.

It was possible that the reduction in robustness of FAA in 12L:12D compared to 18L:6D was due to different phases of food availability relative to the light-dark cycle. Thus, we next shifted the light-dark cycle relative to the time of restricted feeding while mice stayed in their respective free- or locked-wheel conditions in 12L:12D. We shifted the light-dark cycle four times to achieve restricted feeding at three phases: late day (ZT7–11), mid-day (ZT4–8), and early day (ZT1–5). Even though FAA was more robust during late day and mid-day restricted feeding compared to early day feeding, FAA was never as robust as in 18L:6D (Figures 4A,B,D,E). Therefore, the phase of restricted feeding did not account for the reduced robustness of FAA in 12L:12D.

### Nocturnal Activity During Restricted Feeding is Increased in Wheel-Running Conditions

In all experiments in which mice were housed with freely rotating wheels during restricted feeding, nighttime activity increased (Figures 1C,F, 2C, 3C,F, 4C,F). However, nocturnal activity did not increase in cages with locked wheels even if mice had been previously exposed to wheel-running (Figures 3C,F). These data suggest that FAA (output of the food-entrainable oscillator) and nocturnal activity (output of the SCN) are modulated by different mechanisms.

## Discussion

Voluntary wheel-running is not only physical exercise but also a highly rewarding stimulus for many animal species (Sherwin, 1998; Novak et al., 2012; Morgan et al., 2015). Rodents ran greater distances in wheels than on treadmills (Sherwin, 1998). Moreover, rodents worked to unlock running wheels (Kagan and Berkun, 1954; Collier and Hirsch, 1971; Iversen, 1993; Belke, 1997) and developed conditioned place preferences for contexts associated with wheel-running (Belke and Wagner, 2005). Numerous studies have shown that voluntary wheel-running induced molecular and neuronal changes in the brainstem, hypothalamus, and basal ganglia (Morgan et al., 2015). These responses are not limited to rodents in laboratory settings; wheels placed in the natural environment were readily used by wild animals (Meijer and Robbers, 2014).

Although FAA in rodents has been primarily studied in the laboratory, it has been suggested that it represents an activated foraging drive (Mather, 1981). Temporal organization of foraging behaviors is likely critical for the survival of many species (e.g., to increase chances of obtaining food while evading predation). Thus, one can speculate that associating foraging with reward ensures that animals are highly motivated to perform this behavior. Support for this hypothesis comes from several studies linking the dopaminergic reward system to FAA expression (Webb et al., 2009; Mendoza and Challet, 2014). For example, FAA was attenuated when the core of the nucleus accumbens was chemically lesioned in rats (Mendoza et al., 2005b). In addition, FAA was attenuated by inhibition of dopaminergic signaling via treatment with D1 or D2 receptor antagonists (Liu et al., 2012) and in D1 receptor knockout mice (Gallardo et al., 2014; Michalik et al., 2015). It has also been reported that mu-opioid receptor knockout mice display attenuated FAA when feeding is restricted to 3 h during the night (Kas et al., 2004), suggesting that endogenous opioids influence the robustness of FAA.

The current study supports the hypothesis that FAA is intricately linked to reward signaling. We found that wheel-running activity enhanced FAA. The running wheel is rewarding to rodents and known to activate the dopaminergic system and influence endogenous opioids in their brains (Sherwin, 1998; Novak et al., 2012; Morgan et al., 2015). It has been shown that the rewarding effect of wheel running is influenced by endogenous opioids (Lett et al., 2001). The enhanced FAA we observed was not simply due to greater activity levels during wheel running because FAA was similarly enhanced by exposure to locked wheels, as long as the mice had been previously exposed to wheel-running. These data suggest that through operant conditioning, whereby the mice associate the intrinsically rewarding properties of running with the wheel, rewarding value is imparted on the locked wheel. Future studies should explore the putative link between FAA expression and the reward system via direct activation or inhibition of reward circuits.

Dopamine in the brain is also regulated by photoperiod in rodents. In short days, the number of hypothalamic tyrosine hydroxylase-positive neurons declined in male grass rats (Deats et al., 2015). In contrast, in long days, dopamine was elevated in the hypothalamus of C57BL/6J mice (Goda et al., 2015). Previously, our studies demonstrated that mice in long days had more robust FAA compared to short days (Figures 3, 4; Pendergast et al., 2009). A potential caveat of our initial finding was that the differences in phases of restricted feeding relative to the light-dark cycle in different photoperiods could account for the difference in FAA expression. In this study, we eliminated this caveat and showed that regardless of feeding time, FAA is less robust in short photoperiods compared to long photoperiods.

Recently, Dattolo et al. (2016) measured FAA in C57BL/6J mice with or without running discs. With running discs, mice had elevated FAA and reduced nighttime activity so they concluded there was a compensation effect (the total activity each day remained the same but was differentially distributed depending on the presence of the disc). In contrast, we found that both FAA and nocturnal activity were elevated upon exposing mice to free wheels. However, FAA, but not nocturnal activity, was elevated in wheel-experienced mice during exposure to locked wheels. In both studies, passive IR detectors were used to measure general activity; therefore, we do not know what factors accounted for the different results. It is possible that running discs and running wheels have distinct effects on nocturnal activity.

Although FAA was originally described by Richter (1922), relatively little progress has been made in understanding its underlying mechanisms. This is in part due to the elusive nature of the food-entrainable oscillator that controls FAA (the anatomical locus of the food entrainable oscillator is unknown; Stephan, 2002; Davidson, 2009; Mistlberger, 2009). We do not know whether the putative dopaminergic reward signaling that causes the wheel-running enhancement of FAA directly modifies the food-entrainable oscillator itself or acts downstream of the oscillator by regulating its outputs. However, our study is another step toward elucidating the molecular and anatomical substrates of FAA.

## Author Contributions

SY conceived the experiments. DEFLF, CNB, LJ and SY designed and performed the experiments. DEFLF and SY analyzed the data. DEFLF, CNB and SY wrote the manuscript.

## Funding

This research was supported by a National Science Foundation grant IOS-1419477 to SY. DEFLF was supported by Fundação de Amparo à Pesquisa do Estado de São Paulo (FAPESP) process 2013/24740-3.

## Conflict of Interest Statement

The authors declare that the research was conducted in the absence of any commercial or financial relationships that could be construed as a potential conflict of interest.
