# Supplementary material for: The Running Wheel Enhances Food Anticipatory Activity: An Exploratory Study
Source: Front Behav Neurosci. 2016 Jul 5;10:143. doi: 10.3389/fnbeh.2016.00143 (PMC4932273; doi:10.3389/fnbeh.2016.00143)
Supplement: Supplementary file 1 [file SupplementaryMaterial.pdf]

*Supplementary Material*

# The Running Wheel Enhances Food Anticipatory Activity: An Exploratory Study

**Danilo E. F. L. Flôres, Crystal N. Bettilyon, Lori Jia and Shin Yamazaki***

*** Correspondence:** Shin Yamazaki, shin.yamazaki@utsouthwestern.edu

## 1.1 Supplementary Table

**Supplementary Table 1. Summary of statistics.**

| | Photoperiod | Cage type prior to RF | FAA Locked Wheel | FAA Free Wheel | FAA No Wheel |
|---|---|---|---|---|---|
| Experiment 1 | 18:6 | No Wheel | ND | 1,692 ± 678 [a] (n=5) | 501 ± 296 [b] (n=5) |
| Experiment 2 | 18:6 | Free Wheel | ND | 1,401 ± 753 [a] (n=5) | 492 ± 364 [b] (n=5) |
| Experiment 3* | 18:6 | No Wheel | 471 ± 290 (n=5) [a] | 1,066 ± 381 [b] (n=5) | 345 ± 142 [a] (n=5) |
| Experiment 4 | 18:6 | Free Wheel | 2,791 ± 801 [a] (n=5) | 3,040 ± 694 [a] (n=5) | 1,442 ± 415 [b] (n=5) |
| Experiment 5 | 12:12 | Free Wheel | 602 ± 302 [a] (n=6) | 706 ± 155 [a] (n=6) | ND |

The mean ± standard deviation is indicated. * Mouse #206 was excluded based on the criteria > $Q_3$ + 1.5 (IQR). The Wilcoxon signed-rank test (Exp. 1), paired two-tailed t-test (Exp. 2 and Exp. 5), or repeated measures ANOVA followed by Tukey's post-hoc test (Exp. 3 and Exp. 4) was used to compare the robustness of FAA in different cage types within each experiment. The different letters across each experiment indicate significant differences ($p<0.05$). Because the experimental conditions in each experiment were slightly different, we did not compare FAA between experiments. Exp. 1 and Exp. 2: FAA AUC from Free Wheel and No Wheel 2 were used (see Fig 1). Exp. 3: FAA AUC from Locked Wheel, Free Wheel, and No Wheel 2 were used (See Fig. 2). Exp. 4: FAA AUC from Locked Wheel, Free Wheel 2, and No Wheel in the first group; Locked Wheel, Free Wheel, and No Wheel in the second group were used (See Fig. 3). Exp. 5: FAA AUC from Locked Wheel and Free Wheel 2 in the first group; Locked Wheel and Free Wheel in the second group were used (See Fig 4). ND: not determined.

## 1.2 Supplementary Figures

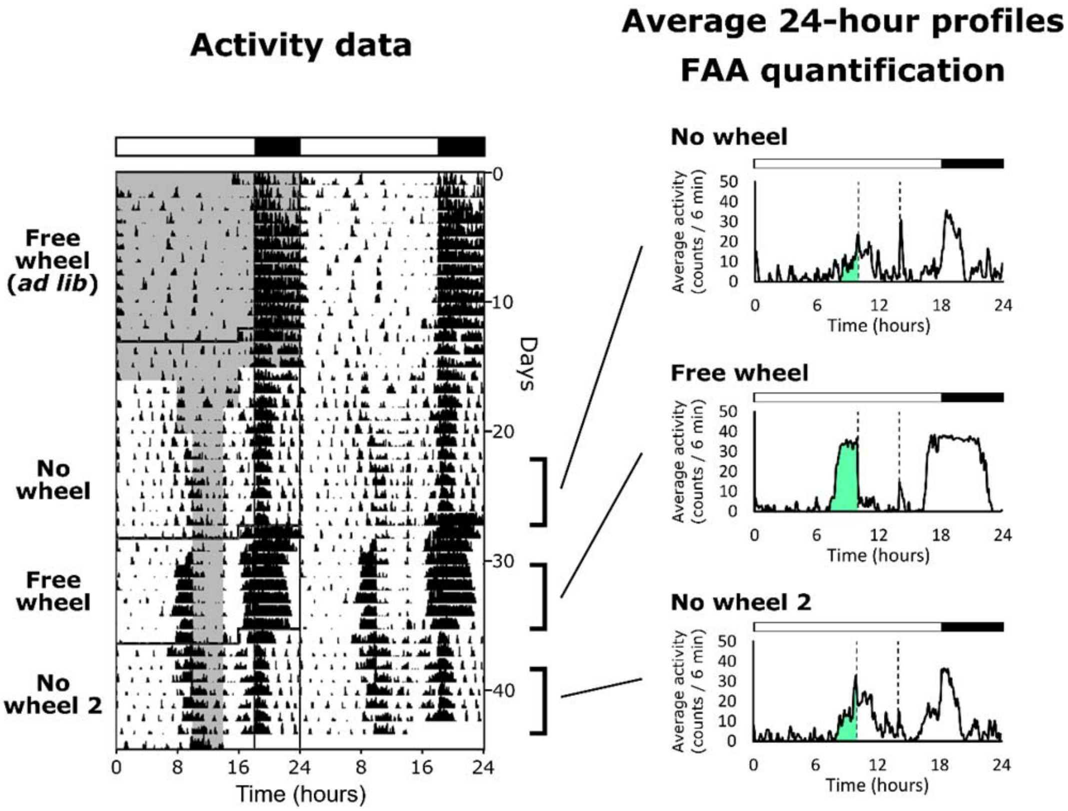


**Supplementary Figure 1. The method for FAA quantification.**

See the materials and methods section for a detailed description.

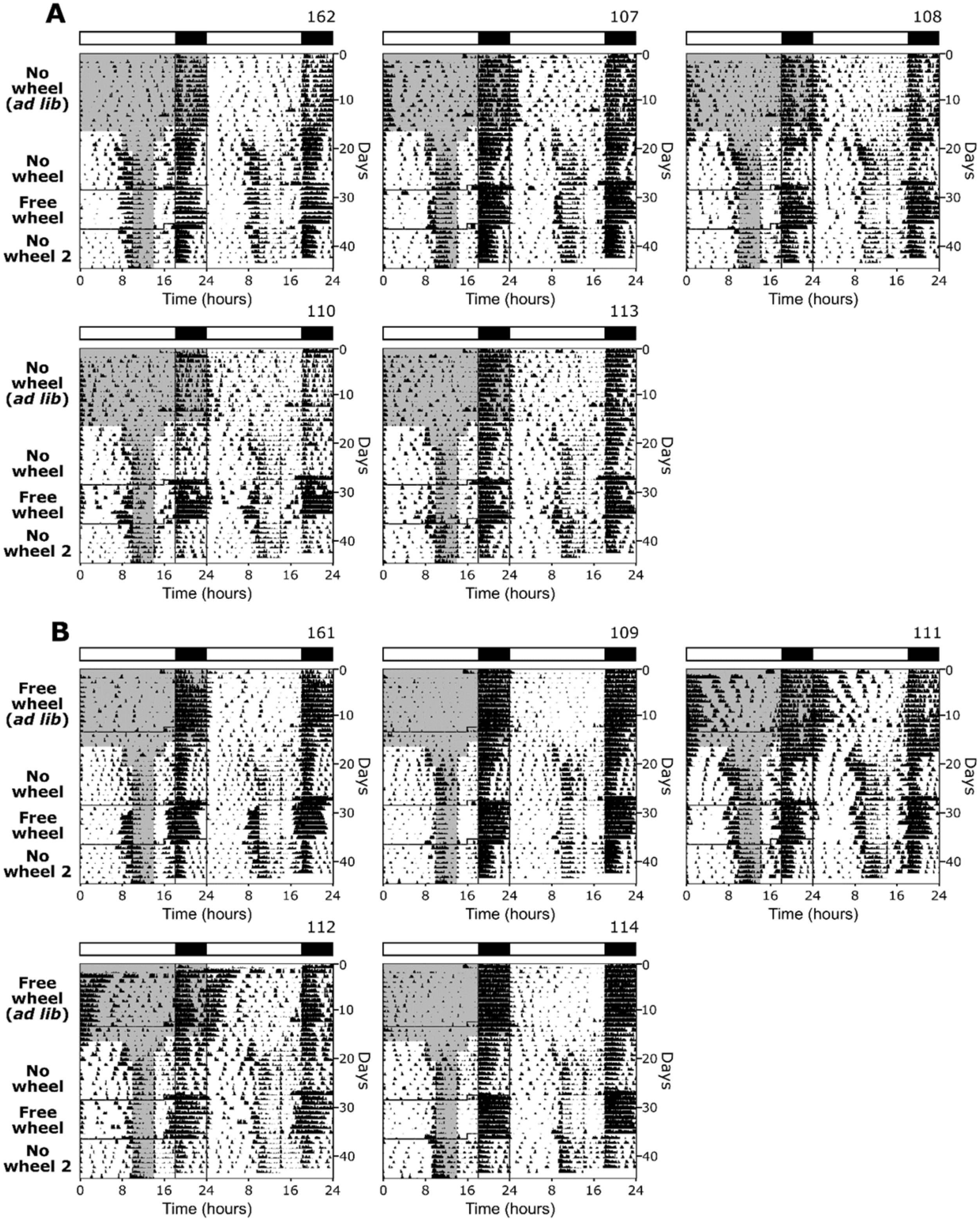


**Supplementary Figure 2. Wheel-running enhances food anticipatory activity irrespective of prior wheel running experience.**

All individual actograms (general activity) are shown.

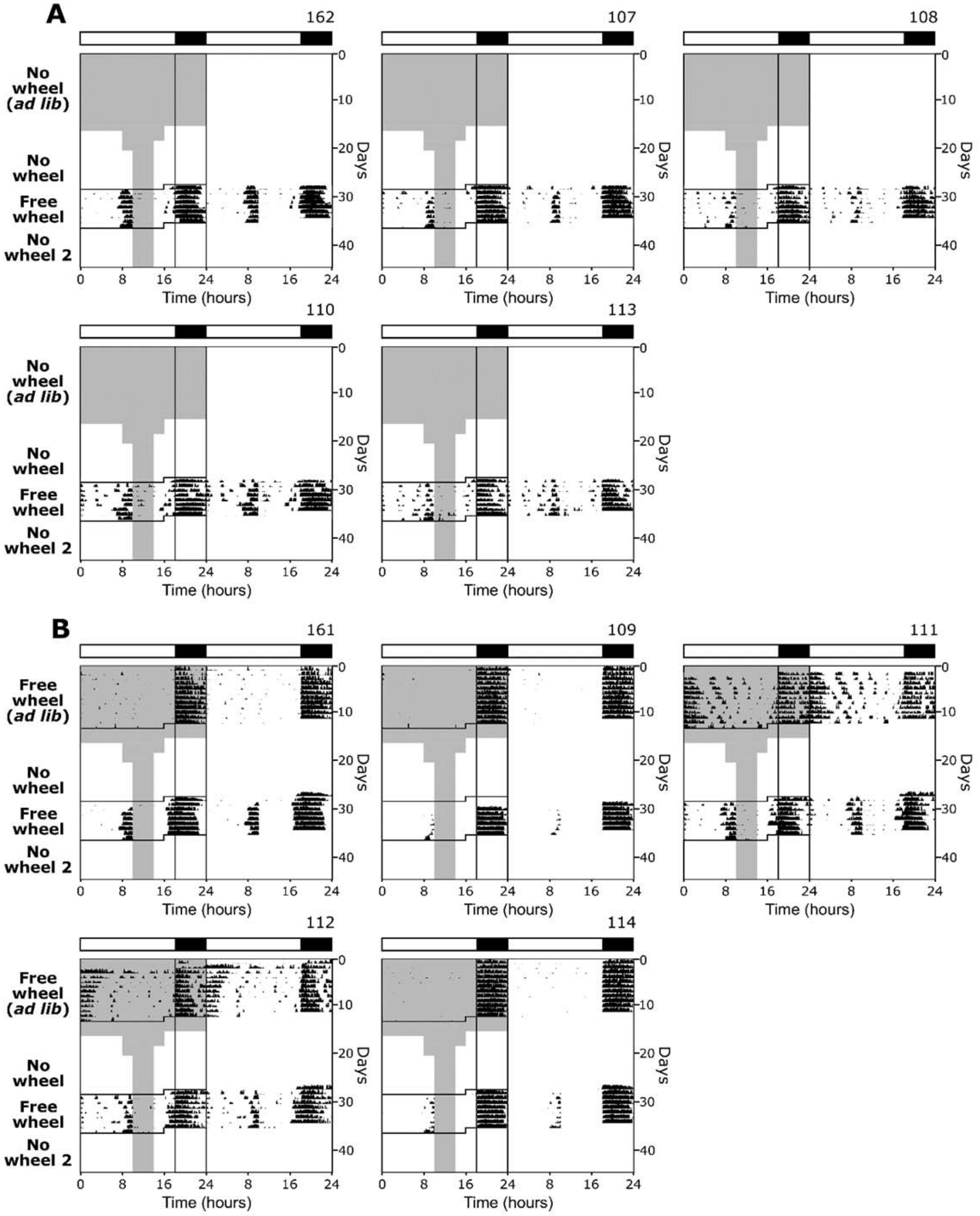


**Supplementary Figure 3. Wheel-running enhances food anticipatory activity irrespective of prior wheel running experience.**

All individual actograms (wheel revolutions) are shown.

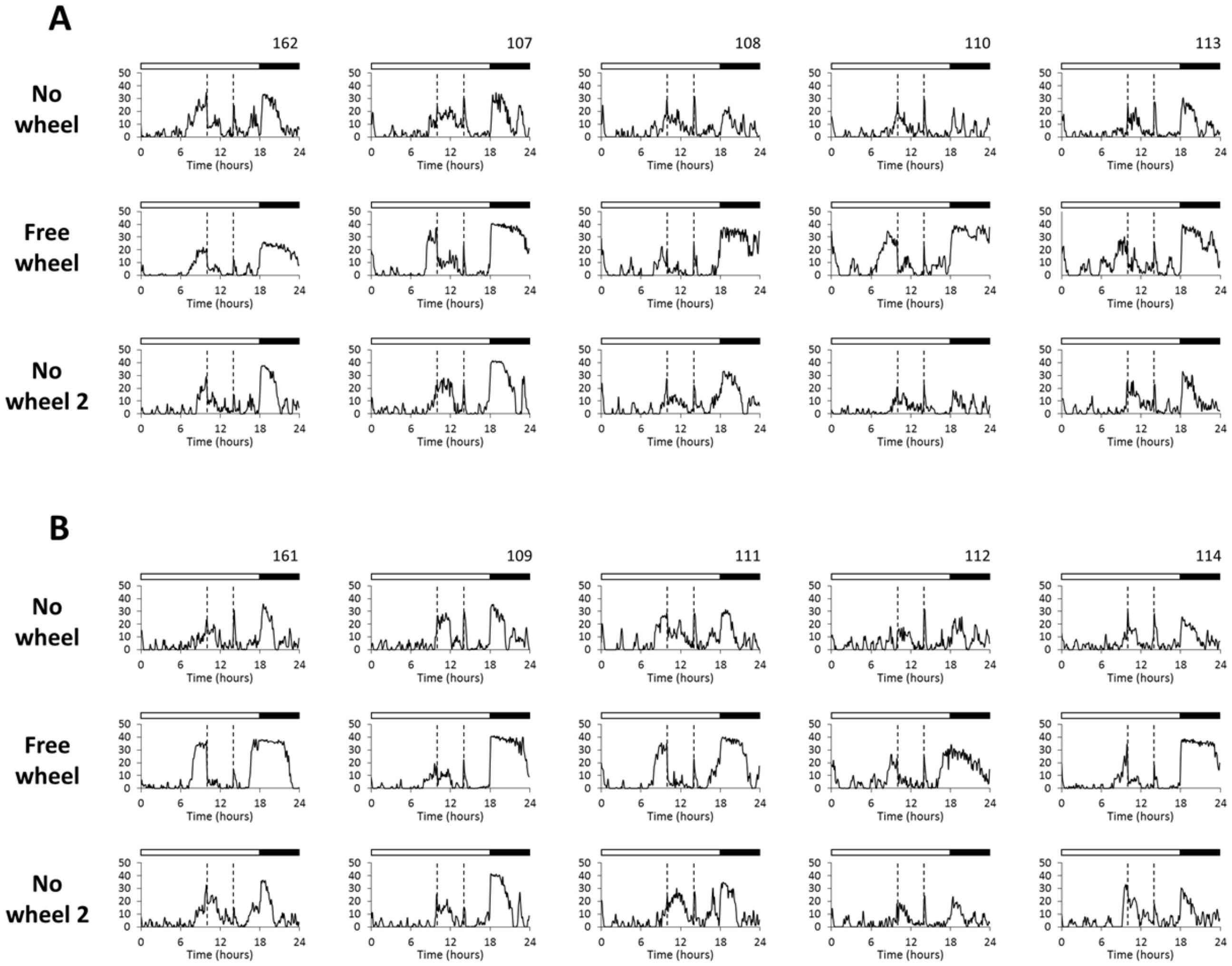


**Supplementary Figure 4. Wheel-running enhances food anticipatory activity irrespective of prior wheel running experience.**

All individual activity profiles are shown.

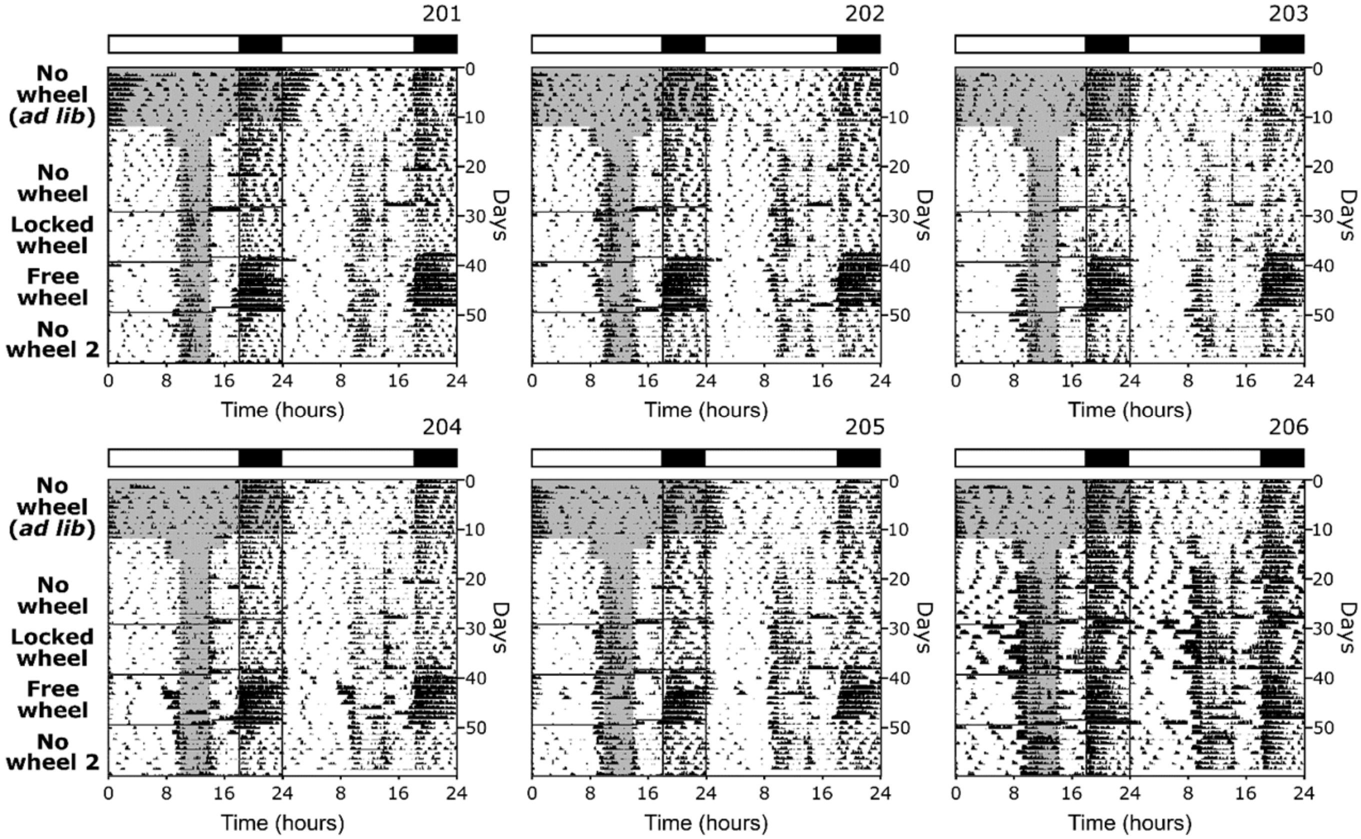


**Supplementary Figure 5. Naïve exposure to a locked running wheel alone does not enhance FAA.** All individual actograms (general activity) are shown.

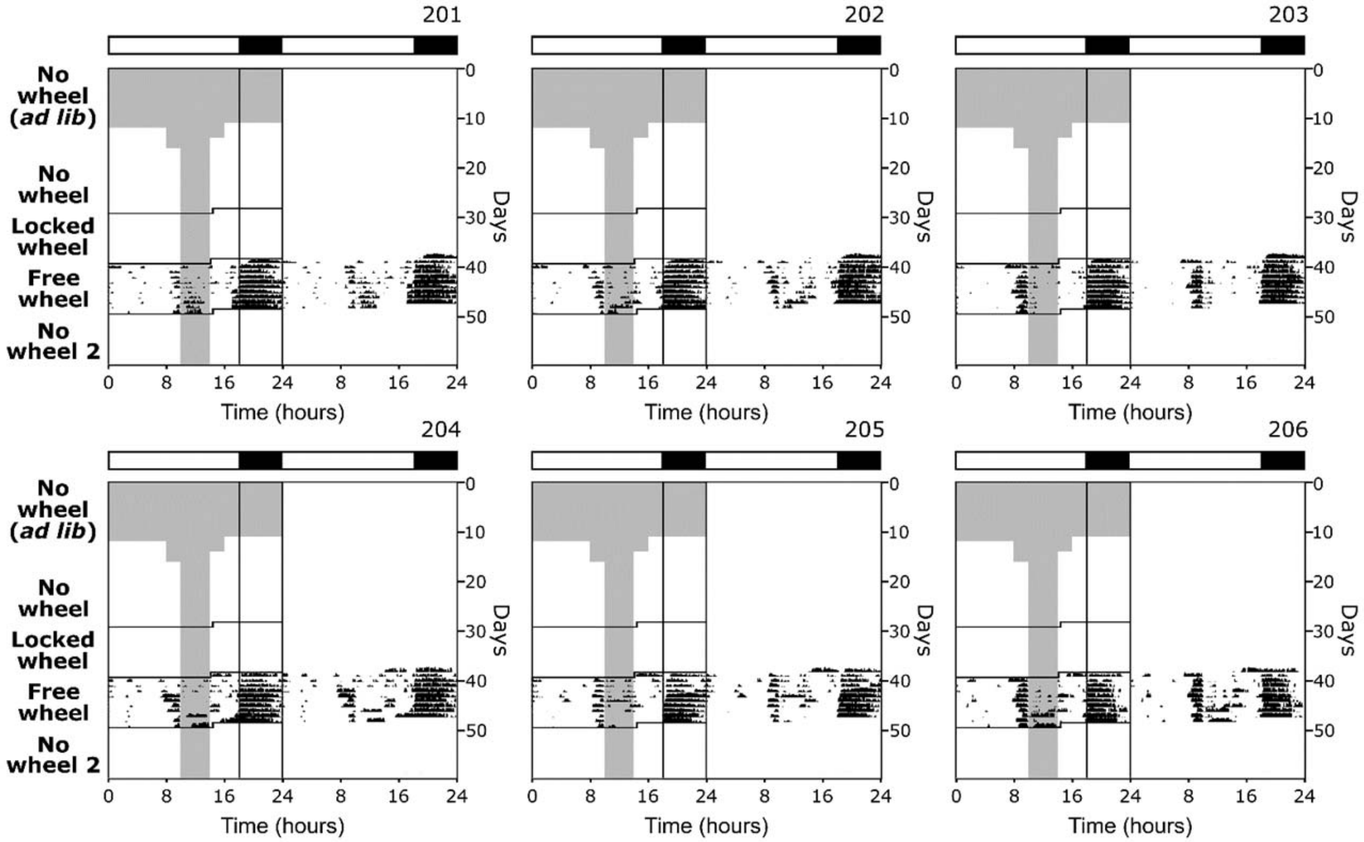


**Supplementary Figure 6. Naïve exposure to a locked running wheel alone does not enhance FAA.** All individual actograms (wheel revolutions) are shown.

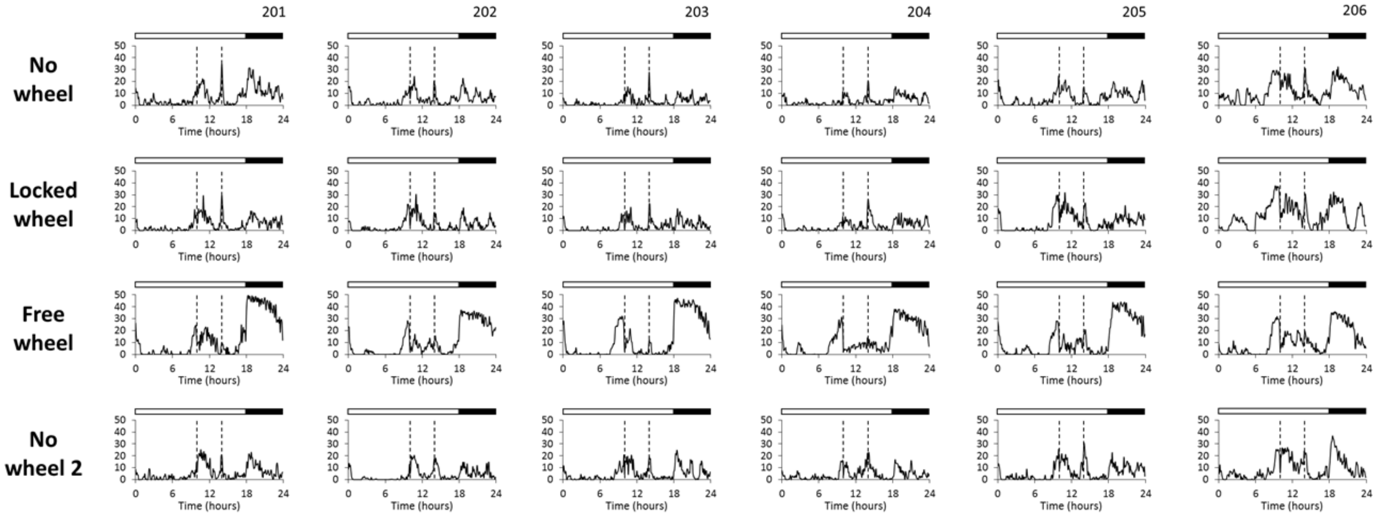


**Supplementary Figure 7. Naïve exposure to a locked running wheel alone does not enhance FAA.** All individual activity profiles are shown.

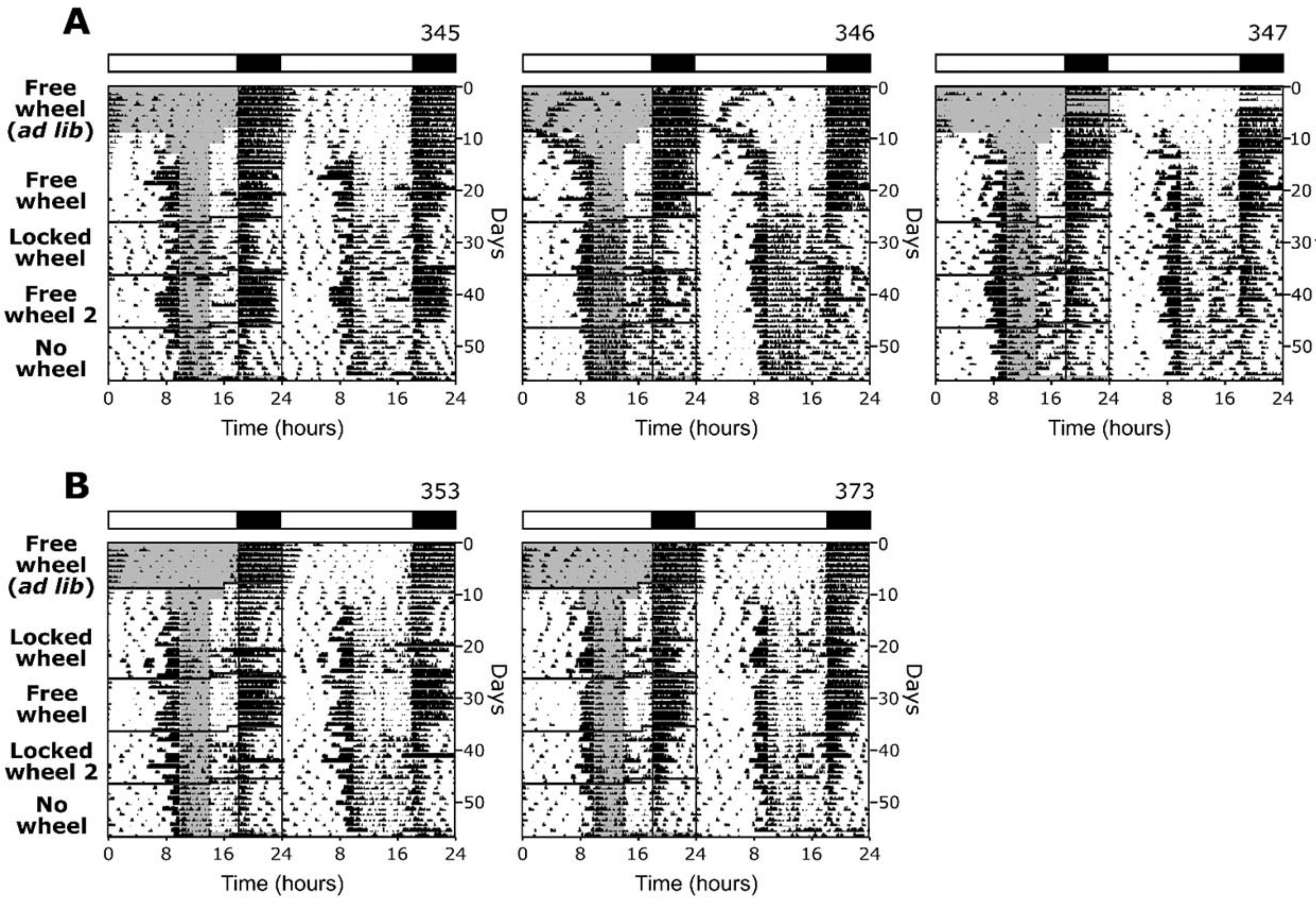


**Supplementary Figure 8. Prior wheel-running experience combined with a locked wheel enhances FAA.** All individual actograms (general activity) are shown.

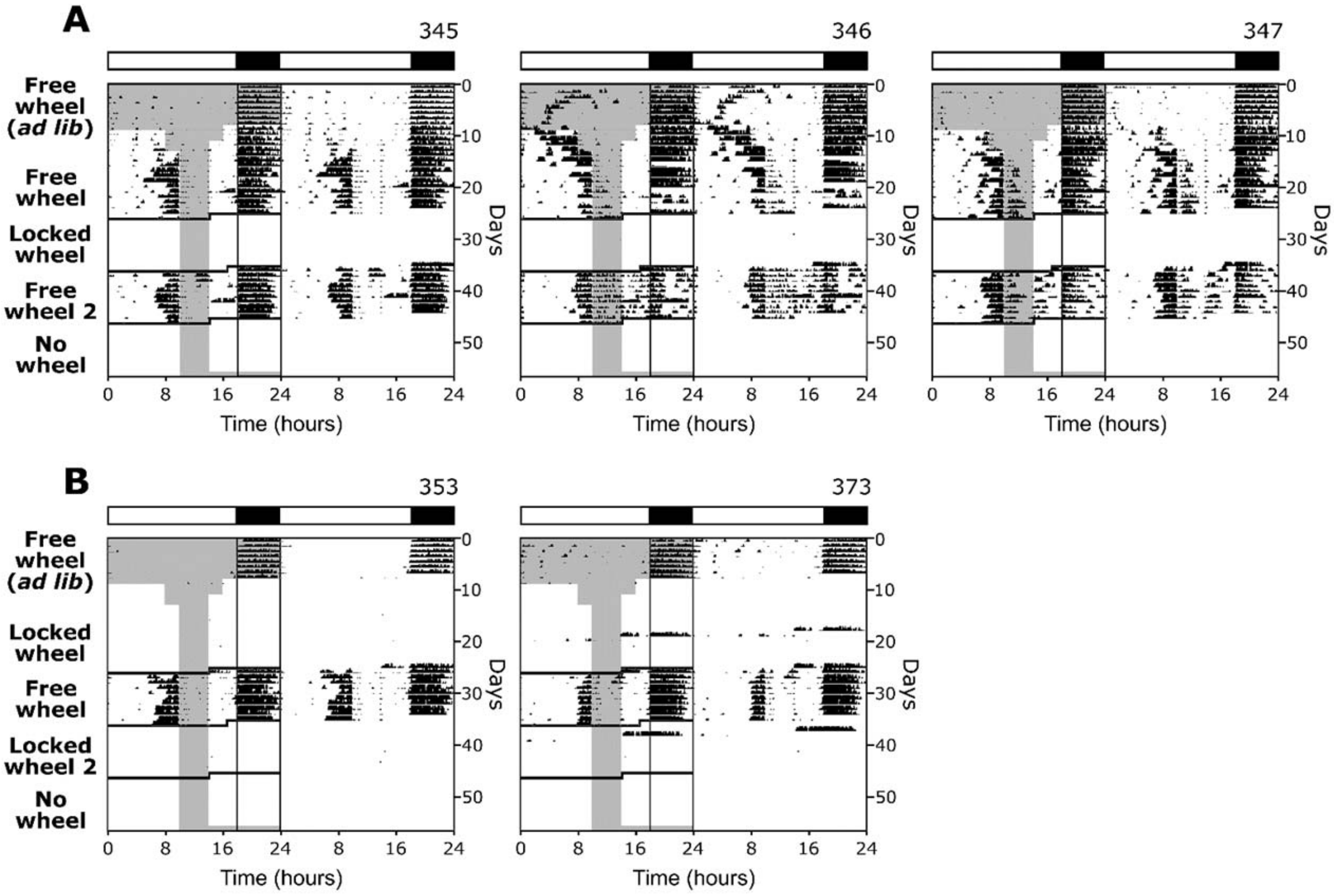


**Supplementary Figure 9. Prior wheel-running experience combined with a locked wheel enhances FAA.** All individual actograms (wheel revolutions) are shown.

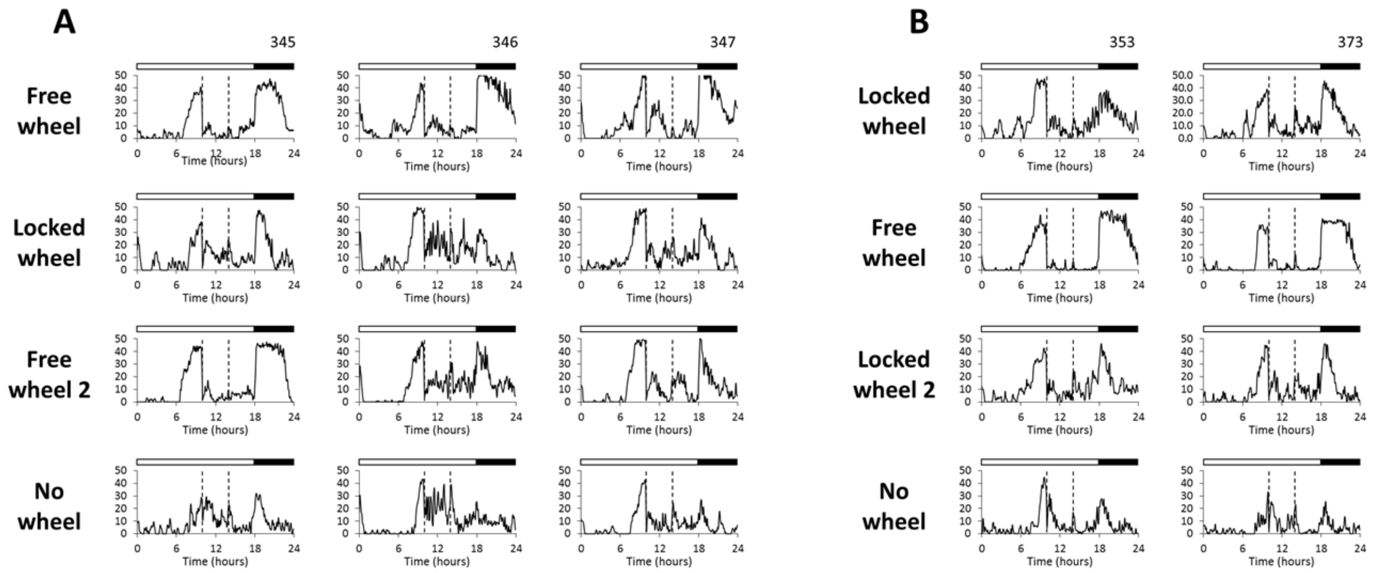


**Supplementary Figure 10. Prior wheel-running experience combined with a locked wheel enhances FAA.**

All individual activity profiles are shown.

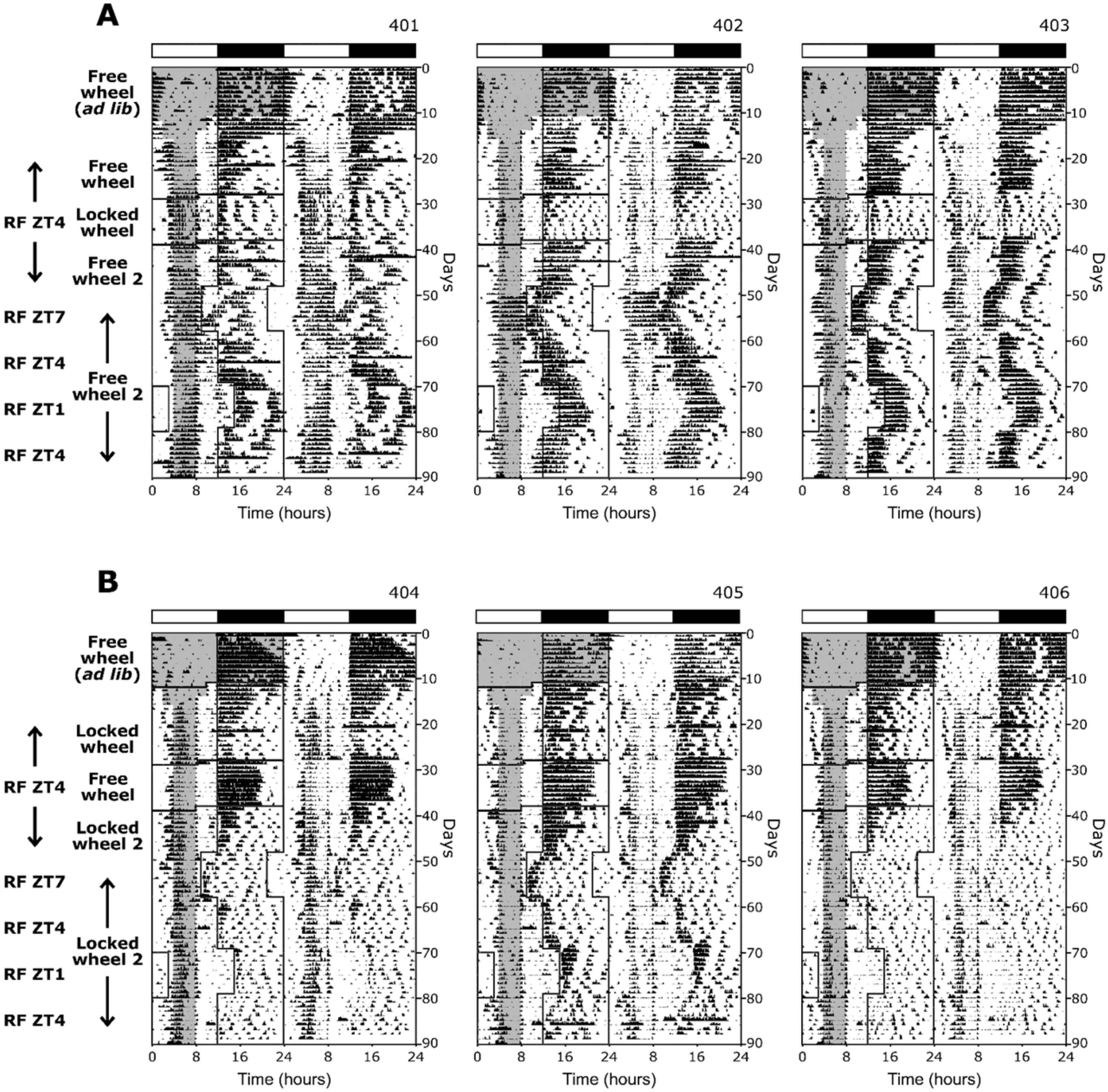


**Supplementary Figure 11. Photoperiod alters the robustness of FAA.**

All individual actograms (general activity) are shown.

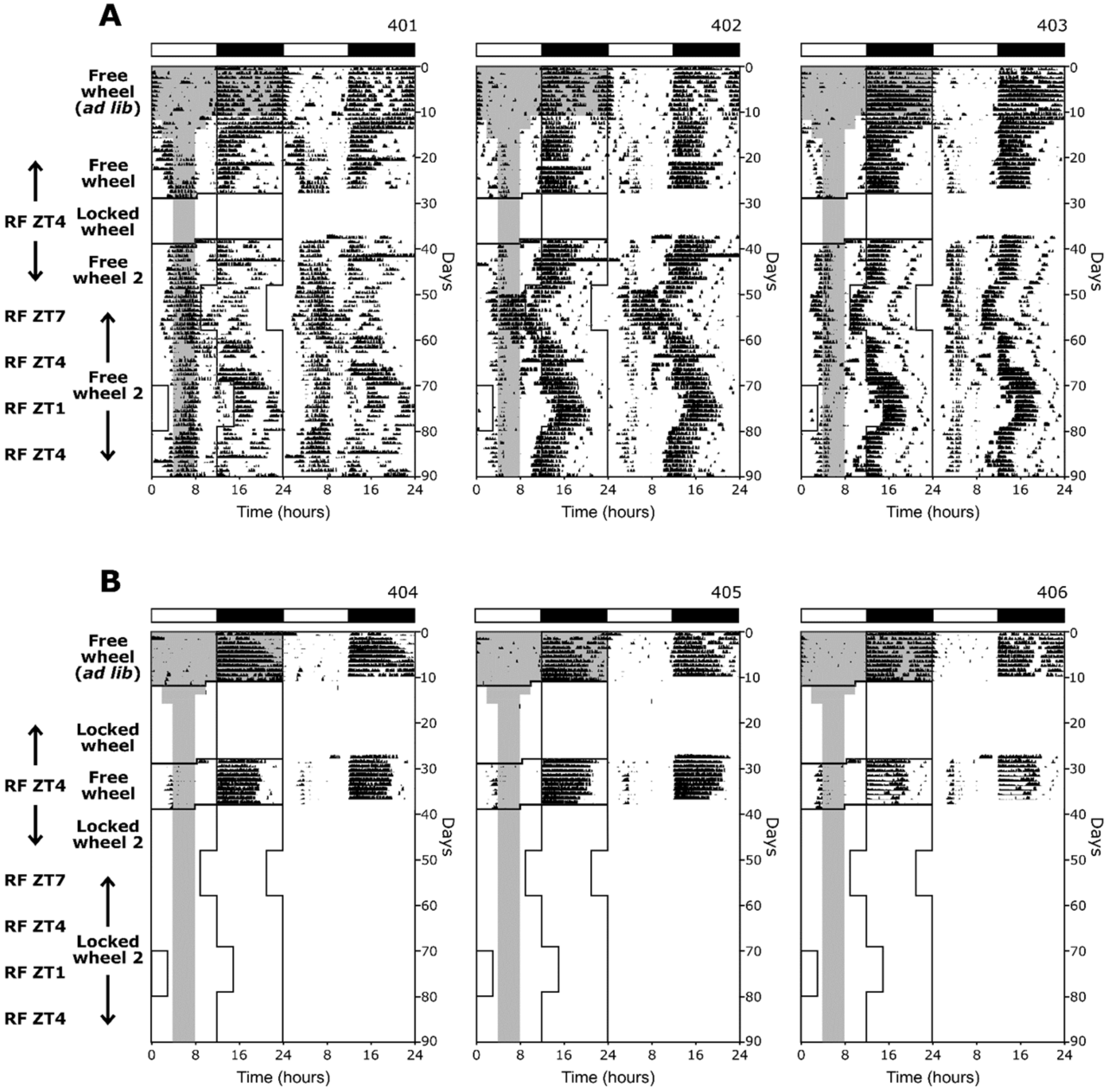


**Supplementary Figure 12. Photoperiod alters the robustness of FAA.**

All individual actograms (wheel revolutions) are shown.

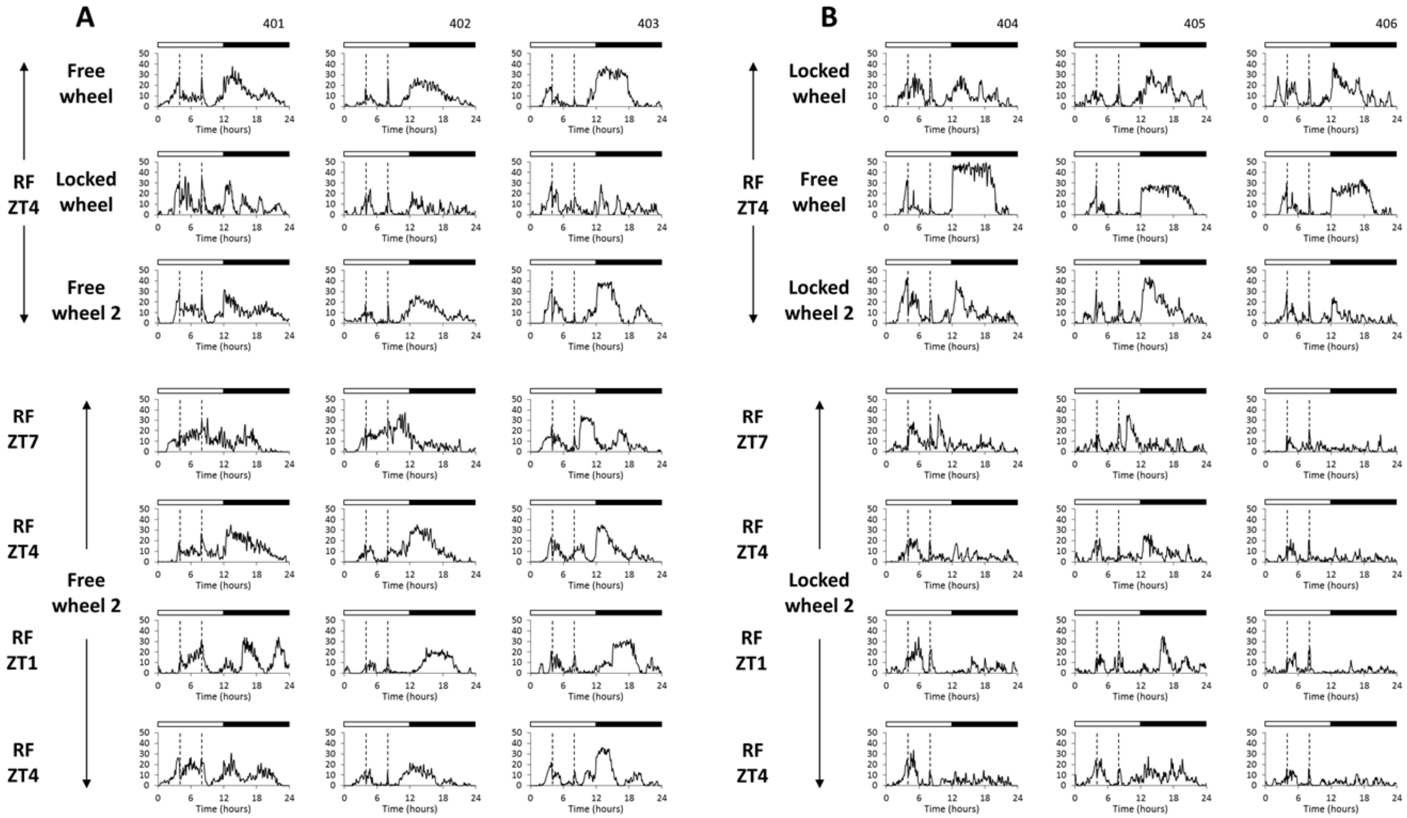


**Supplementary Figure 13. Photoperiod alters the robustness of FAA.**

All individual activity profiles are shown.
